# Advances in chemistry and bioactivity of the genus *Erythroxylum*

**DOI:** 10.1007/s13659-022-00338-z

**Published:** 2022-04-14

**Authors:** Yulian Lv, Tian Tian, Yong-Jiang Wang, Jian-Ping Huang, Sheng-Xiong Huang

**Affiliations:** 1 State Key Laboratory of Phytochemistry and Plant Resources in West China, CAS Center for Excellence in Molecular Plant Sciences, Kunming Institute of Botany, Chinese Academy of Sciences, Kunming, 650201 China; 2 University of Chinese Academy of Sciences, Beijing, 100049 China; 3 State Key Laboratory of Southwestern Chinese Medicine Resources, Innovative Institute of Chinese Medicine and Pharmacy, Chengdu University of Traditional Chinese Medicine, Chengdu, 611137 China

**Keywords:** *Erythroxylum*, Natural products, Phytoconstituent, Bioactivity

## Abstract

*Erythroxylum* P. Browne is the largest and most representative genus of Erythroxylaceae family. It contains approximately 230 species that are mainly distributed in tropical and subtropical regions. Some species in this genus, such as *E.* *monogynum* and *E.* *coca*, have been used as folk medicines in India or South America for a long history. It is well known that *Erythroxylum* plants are rich in tropane alkaloids, and the representative member cocaine shows remarkable activity in human central nervous system. However, many other types of active compounds have also been found in *Erythroxylum* along with the broadening and deepening of phytochemical research. To date, a total of 383 compounds from *Erythroxylum* have been reported, among which only 186 tropane alkaloids have been reviewed in 2010. In this review, we summarized all remained 197 compounds characterized from 53 *Erythroxylum* species from 1960 to 2021, which include diterpenes, triterpenes, alkaloids, flavonoids, and other derivates, providing a comprehensive overview of phytoconstituents profile of *Erythroxylum* plants. In addition, the biological activities of representative phytochemicals and crude extracts were also highlighted.

**Graphical Abstract:**

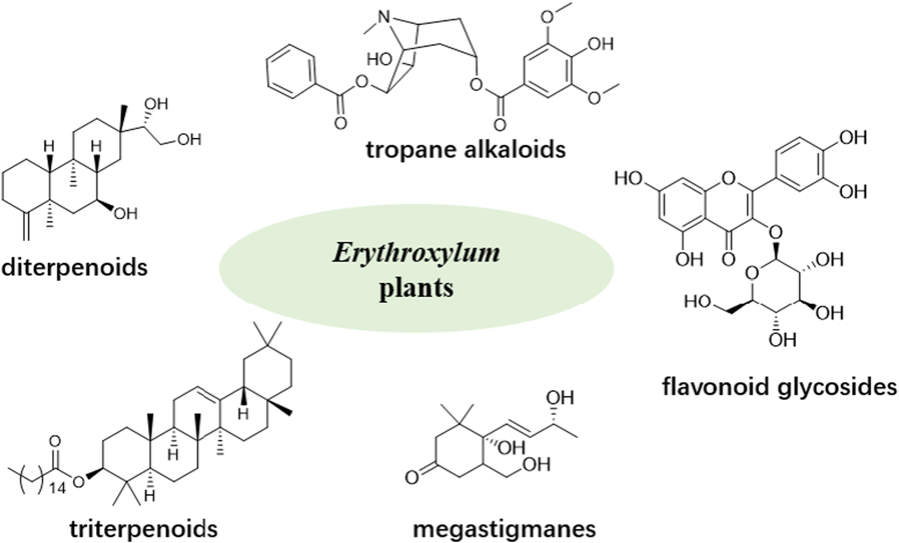

## Introduction

In the long evolutionary process of nature, plants have acquired the ability to synthesize various compounds to better adapt to stimulations in the environment. The accumulation of practical experience has made human realize that these substances are also of significant importance for the treatment of human diseases and the improvement of the quality of life. With the support of technology in compound extraction, separation and structural identification, the active substances in traditional herbs are gradually being discovered by humans. Therefore, modern medicine based on a single or several compounds has been developed. With the deepening of research on plant natural products, new biologically active compounds are constantly being discovered and further applied in medicine, health care and agriculture. Sorting out and summarizing the plant distribution, structure, and activity characteristics of these newly discovered phytocompounds will confer us effective information in rational use of plant resources.

*Erythroxylum* P. Browne, the representative genus of Erythroxylaceae family, is especially well known for its phytoconstituents of tropane alkaloids (TAs), such as cocaine [1, 2]. Species of this genus are mainly distributed in tropical and subtropical regions including South America, South Africa, Southeast Asia and Australian flora [2]. As the largest genus of the Erythroxylaceae family, approximately 230 species are included in *Erythroxylum* [2], among which *E.* *coca* and *E.* *novogranatense* are the famous plant sources of cocaine. Before achieving the purification of cocaine from plants in 1859 [3], the leaves of *E.* *coca* or *E.* *novogranatense* had been chewed by the Indigenous South American as stimulant and hunger-suppressant for over a thousand years*.* The remarkable biological activity of cocaine in human central nervous system attracted widespread attention to compounds in plants of this genus. Accordingly, numerous of cocaine analogs (TAs), as well as other bioactive compounds have been found in *Erythroxylum* [4, 5].

To date, no comprehensive summary on chemical compositions found in *Erythroxylum* species and their bioactivities has been reported, though Oliveira et al. [6] presented an excellent review focusing on structures of TAs isolated from this genus in 2010 and Dr. John D'Auria’ s group discussed application potentials of *Erythroxylum* species worldwide in mental health, nutrition, agriculture, and commercialization based on studies on representative compounds discovered in this genus [7]. Attracted by the diverse biological activity of compounds found in *Erythroxylum*, which included anaesthetic [8], antioxidative [9, 10], anti-inflammatory [9], cytotoxic [11], anticancer [12], and insecticidal activities [13], as well as neutralization of snake venom [14], we therefore aimed to provide a comprehensive review of all compounds reported in *Erythroxylum* species from 1960 to 2021 and an update of alkaloids isolated after 2010 here, which is supposed to be essential for further effective development and utilization of plant resources in the genus in the future. Additionally, we also presented an overview of the biological activities of representative phytochemicals and crude extracts at the end of the review, providing medicinal and commercial application prospects of *Erythroxylum* species.

## Chemical composition

Based on the published results dedicated to study chemical composition of *Erythroxylum* species, 383 compounds, including diterpenes, triterpenes, flavonoids, alkaloids, and other derivates, have been found in 67 *Erythroxylum* species. Among these, 186 TAs compounds identified in *Erythroxylum* plants before 2010 have been systematically reviewed by Oliveira et al. [6]. Therefore, here we summarized all remained 197 compounds characterized from 53 *Erythroxylum* species from 1960 to 2021, which include diterpenes, triterpenes, alkaloids, flavonoids, and other derivates.

According to the literature, *Erythroxylum* plants are rich in alkaloids. Especially *E.* *coca*, *E.* *coca* var*.* *coca*, and *E.* *novogranatense* var. *novogranatense*, the content of total alkaloids varies from 0.5% to 2.4% in leaves (dry mass, Table 1) [15]. Particularly, high cocaine content (0.13%-0.76% dry mass) was found in *E.* *coca* and *E.* *novogranatense* leaves [16]. In 2006, Stefan Bieri et al. [17] analyzed the cocaine distribution in 51 plant species and cocaine was detected only in 23 *Erythroxylum* species with the content less than 0.001% (dry leaves). High production of total phenols, total tannins and total flavonoids of up to 17.97%, 8.4%, and 3.87% (dry leaves), respectively, was reported in *E.* *suberosum*, *E.* *tortuosum*, and *E.* *deciduum* [18] (Table 1). Additionally, the total diterpenes content determined in stems of *E.* *australe* and *E.* *pictum* ranged from 0.09% to 1.8% (dry mass, Table 1).

**Tabel 1:** The content of principal components in several *Erythroxylum* species

Species	Total alkaloids (dry leaves) (%)	Total phenols (dry leaves) (%)	Total tannins (dry leaves) (%)	Total flavonoids (dry leaves) (%)	Total diterpenes (dry stems) (%)	Refs.
*E.* *coca*	0.5–1.5					[15]
*E.* *coca* var. *coca*	1.05–2.26					[19]
*E.* *novogranatense* var. *novogranatense*	1.4–2.4					[19]
*E.* *suberosum*		17.97	6.31	3.87		[18]
*E.* *tortuosum*		10	8.4	0.064		[18]
*E.* *deciduum*		12.04	0.87	1.37		[18]
*E.* *australe*					1.8	[5]
*E.* *pictum*					0.09–1.1%	[20]

### Diterpenes

**Fig. 1:**
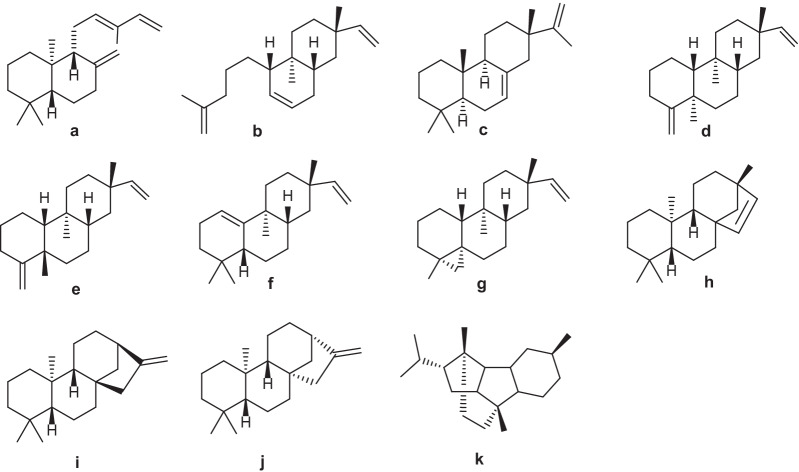
Skeletons of diterpenes found in *Erythroxylum* plants

Plants of *Erythroxylum* are rich in diterpenoids, which have been extensively studied since the last century. In particular, Connolly [21–24] and Kapadi [25–27], who focused on investigating diterpenoids of *E.* *monogynum* in 1960s, provided the earliest knowledge of diterpenoids in *Erythroxylum* species. Using nuclear magnetic resonance (NMR) spectroscopy and chemical reactions, they and their coworkers elucidated the structures of 17 diterpenoids in *E.* *monogynum*. To date, about 11 types of diterpene skeletons (**a**–**k**) have been identified from plants in this genus (Fig. 1). Based on the number of rings in the diterpene skeletons, diterpenes found in *Erythroxylum* species could be divided into bicyclic diterpenes, tricyclic diterpenes, and tetracyclic diterpenes.

#### Bicyclic diterpenes

**Fig. 2:**
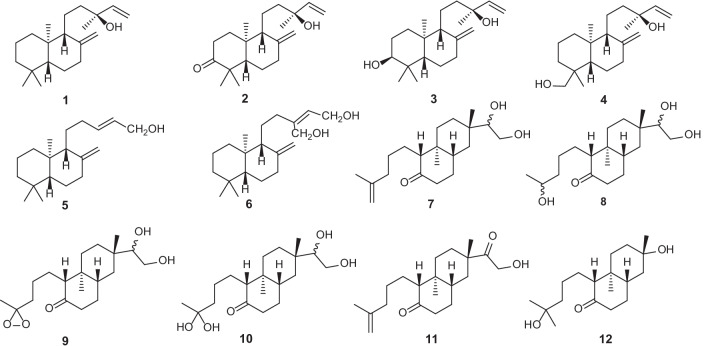
Chemical structures of bicyclic diterpenes (**1–12**) found in *Erythroxylum* plants

Labdane is a typical bicyclic diterpene, which forms the structural skeleton for many diterpene compounds found in plants [28–30]. In *Erythroxylum*, six *ent*-labdane derivatives (**1–6**) have been isolated and characterized from nine species of this genus in the past decades [20, 31, 32] (Table 2). Additionally, Ansell [20] et al. first found six 4,5-*seco*-rosane derivative diterpenoids (**7–12**) from *E.* *pictum* in 1993 (Table 2)*.* Since these derivatives were shown to be characteristic of *E.* *pictum*, they named this novel bicyclic diterpene skeleton, 4,5-*seco*-rosane, as pictane. Later, they found one of these derivatives of pictane, *ent*-15*ξ*,16-dihydroxypictan-4(18)-en-5-one (**7**), was also present in other six species of *Erythroxylum* [31]. The distribution and structures of these bicyclic diterpenes are listed in Table 2 and Fig. 2, respectively.

**Table 2:** Bicyclic diterpenes isolated from *Erythroxylum* plants

No.	Compounds	Plant source	Refs.
**1**	*ent*-labda-8(17),14-dien-13*R*-ol	*E.* *pictum*, *E.* *areolatum*, *E.* *cuneatum*, *E.* *rotundifolium*	[20, 31]
**2**	*ent*-13*R*-hydroxylabda-8(17)-dien-3-one	*E.* *pictum*, *E.* *betulaceum*, *E.* *cuneatum*, *E.* *rotundifolium*	[20, 31, 32]
**3**	*ent*-labda-8(17),14-dien-3*β*,13*R*-diol	*E.* *pictum*, *E.* *betulaceum*, *E.* *cuneatum*, *E.* *delagoense*	[20, 31, 32]
**4**	*ent*-labda-8(17),14-dien-13*R*,18-diol	*E.* *pictum*, *E.* *rotundifolium*	[20, 31]
**5**	*ent*-labda-8(17),13*E*-dien-15-ol	*E.* *pictum*, *E.* *deciduum*, *E.* *zambesiacum*	[20, 31]
**6**	*ent*-labda-8(17),13*E*-dien-15,16-diol	*E.* *argentinum*	[31]
**7**	*ent*-15*ξ*,16-dihydroxypictan-4(18)-en-5-one	*E.* *pictum*, *E.* *areolatum*, *E.* *cuneatum*, *E.* *delagoense*, *E.* *microphyllum*, *E.* *zambesiacum*, *E.* *rotundifolium*	[20, 31]
**8**	*ent*-4,15*ξ*,16-trihydroxypictan-5-one	*E.* *pictum*	[20]
**9**	*ent*-15*ξ*,16-dihydroxy-4,18-epoxypictane-5-one	*E.* *pictum*	[20]
**10**	*ent*-4,15*ξ*,16,18-tetrahydroxypictan-5-one	*E.* *pictum*	[20]
**11**	*ent*-16-hydroxypictan-4(18)-ene-5,15-dione	*E.* *pictum*	[20]
**12**	*ent*-4,13*α*-dihydroxy-15*ξ*,16-bisnorpictan-5-one	*E.* *pictum*	[20]

#### Tricyclic diterpenes

**Fig. 3:**
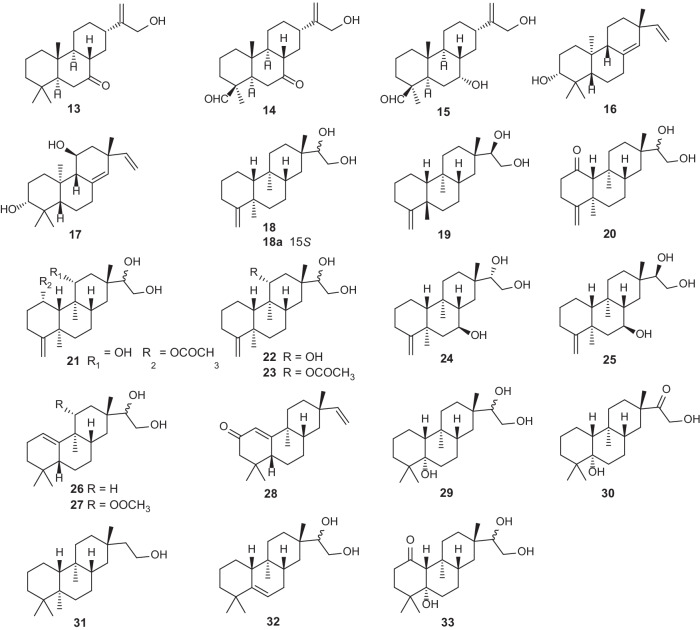
Chemical structures of tricyclic diterpenes (**13–33**) found in *Erythroxylum* plants

21 tricyclic diterpene compounds with four skeleton types (abietane, pimarane, dolarbrane, and rosane) have been isolated from *Erythroxylum* genus (Fig. 3; Table 3). Among these compounds, there are three abietane (**13–15**) [33] and two pimarane diterpenoids (**16–17**) [31] obtained from *E.* *suberosum* and *E.* *cuneatum*, respectively. Dolarbrane-type diterpene was first found in the leaves of *Thujopsis* *dolabrata* of Cupressaceae in 1964 [34]. Almost at the same time, Connolly [21], who focused on the phytochemistry of *E.* *monogynum*, characterized erythroxydiol Y (**18**) from this *Erythroxylum* plant. In 1993, seven new dolarbrane-type derivatives (**19–25**) were reported by Ansell et al. [20, 31]. In addition, they identified seven rosane-type (**26–32**) diterpenoids from several *Erythroxylum* species. Another rosane-type compound (**33**) was found in *E.* *barbatum* by dos Santos [35].

**Table 3:** Tricyclic diterpenes isolated from *Erythroxylum* plants

No.	Compounds	Plant source	Refs.
**13**	7-oxo-16-hydroxy-abiet-15(17)-en-19-al	*E.* *suberosum*	[33]
**14**	7-oxo-abiet-15(17)-en-16-ol	*E.* *suberosum*	[33]
**15**	7*α*,16-dihydroxy-abiet-15(17)-en-19-al	*E.* *suberosum*	[33]
**16**	*ent*-pimara-8(14),15-dien-3*α*-ol	*E.* *cuneatum*	[31]
**17**	*ent*-3*α*,11*β*-dihydroxypimara-8(14),15-diene	*E.* *cuneatum*	[31]
**18**	erythroxydiol Y (allodevadarool)	*E.* *monogynum*	[5, 21, 22, 36]
**18a**	*ent*-dolabr-4(18)-ene-15*S*,16-diol	*E.* *pictum,* *E.* *argentinum,* *E.* *delagoense,* *E.* *macrocarpum,* *E.* *rotundifolium,* *E.* *sideroxyloides*	[20, 31]
**19**	*ent*-5*β*-dolabr-4(18)-ene-15*R*,16-diol	*E.* *pictum*	
**20**	*ent*-15,16-dihydroxydolabr-4(18)-en-1-one	*E.* *sideroxyloides*	[31]
**21**	*ent*-1*α*-acetoxydolabr-4(18)-ene-11*α*,15*ξ*,16-triol	*E.* *sideroxyloides*	[31]
**22**	*ent*-dolabr-4(18)-ene-11*α*,15*ξ*,16-triol	*E.* *macrocarpum,* *E.* *sideroxyloides*	[31]
**23**	*ent*-11*α*-acetoxydolabr-4(18)-ene-15*ξ*,16-diol	*E.* *macrocarpum,* *E.* *sideroxyloides*	[31]
**24**	*ent*-dolabr-4(18)-ene-7*β*,15*S*,16-triol	*E.* *sideroxyloides,* *E.* *pictum,*	[20, 31]
**25**	*ent*-dolabr-4(18)-ene-7*β*,15*R*,16-triol	*E.* *pictum*	[20]
**26**	*ent*-5*β*-ros-1(10)-en-15*ξ*,16-diol	*E.* *zambesiacum*	[31]
**27**	*ent*-11*α*-acetoxy-5*α*-ros-1(10)-en-15*ξ*,16-diol	*E.* *zambesiacum*	[31]
**28**	*ent*-2-oxo-ros-1(10),15-diene	*E.* *zambesiacum*	[31]
**29**	*ent*-rosane-5*α*,15*ξ*,16-triol	*E.* *cuneatum,* *E.* *areolatum,* *E.* *sideroxyloides,* *E.* *pictum,* *E.* *zambesiacum*	[20, 31]
**30**	*ent*-5*α*,16-dihydroxyrosan-15-one	*E.* *pictum*	[20]
**31**	*ent*-rosane-5*α*,16-diol	*E.* *pictum*	[20]
**32**	*ent*-ros-5-en-15*ξ*,16-diol	*E.* *pictum*	[20]
**33**	*ent*-rosan-1-one-5*β*,15*ξ*,16-triol	*E.* *barbatum*	[35]

#### Tetracyclic diterpenes

**Fig. 4:**
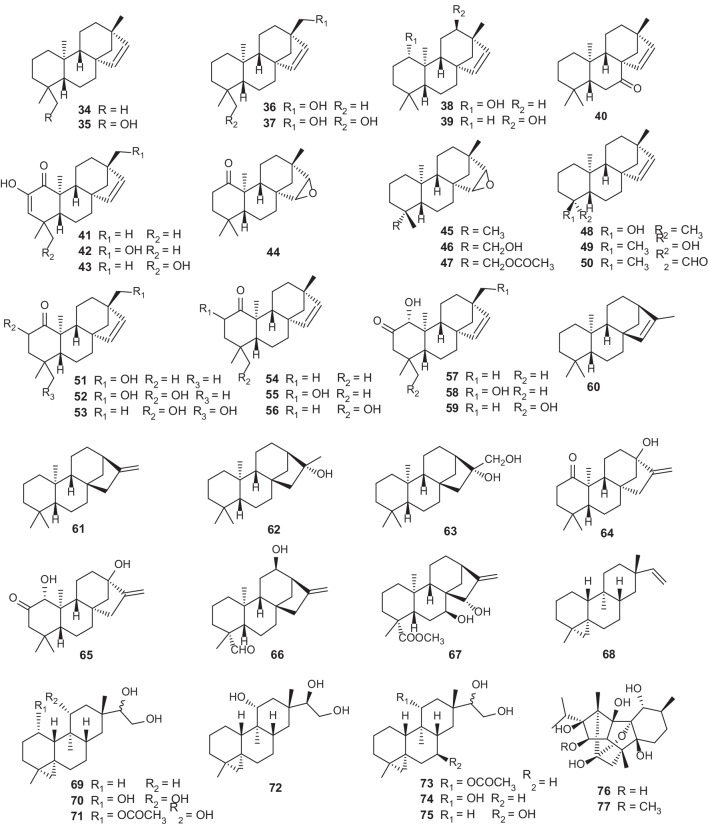
Chemical structures of tetracyclic diterpenes (**34–77**) found in *Erythroxylum* plants

*Erythroxylum* is a prolific source of beyerene diterpenes [5, 24, 31]. More than 20 beyerene derivatives (**34–59**) have been identified from nine *Erythroxylum* species [5, 24, 25, 31, 37–40], while diterpenoids isolated from *E.* *australe* consisted preponderantly of beyerene derivatives [5, 24] (Fig. 4; Table 4). Importantly, in a recent research, auto-oxidation of the aldehyde group of *ent*-beyer-15-en-19-al (**50**) isolated from *E.* *monogynum* to a carboxylic acid group was observed, and this auto-oxidation could take place both with and without the concurrent epoxidation of the 15,16-double bond, indicating that some beyerene type diterpenoids identified previously may be artefacts arising from the auto-oxidation reaction [40]. Tetracyclic diterpene *ent*-kaurene is a critical intermediate in gibberellin hormones biosynthesis pathway in plants, and kaurene diterpenes are widely distributed in nature. Seven kaurene diterpenes (**61–67**) have been isolated and identified from *Erythroxylum* plants [5, 20, 31, 33, 41] (Fig. 4; Table 4), among which erythroxylisin A (**64**) and erythroxylisin B (**65**) obtained from roots of *E.* *barbatum* are unusual kaurene diterpenes with a *cis*-orientation of the C-20 methyl and the CH_2_-15 methylene groups [41]. Devadarane is a tetracyclic diterpene skeleton completely different from the two mentioned above (Fig. 4; Table 4). Devadarane-type diterpene compounds (**68–75**) were first discovered in *E.* *monogynum* [21, 42]. However, the structure of triol Q (**72**) was not determined until McCrindle. R [43] undertook an X-ray analysis two years later. Although devadarane-type diterpenes have been identified in five species of this genus, only eight devadarane derivatives (**68–75**) have been reported so far (Fig. 4; Table 4). Ryanodane diterpenes (**76–77**) [13] were originally isolated from ripe fruits of *E.* *passerinum*, and later ryanodanol (**76**) was also identified in *E.* *nummularia* leaves (Fig. 4; Table 4). This type of diterpenoids has a complicated skeleton. According to reports in the literature since 1960, only two compounds (**76–77**) of this type have been discovered in the genus *Erythroxylum*.

**Table 4:** Tetracyclic diterpenes isolated from *Erythroxylum* plants

No.	Compounds	Plant source	Refs.
**34**	*ent*-beyer-15-ene (( +)-hibaene)	*E.* *australe*, *E.* *monogynum*, *E.* *zambesiacum*	[5, 25, 31, 39]
**35**	*ent*-beyer-15-en-19-ol (erythroxylol A)	*E.* *australe*, *E.* *zambesiacum*, *E.* *monogynum*	[5, 31, 37, 38]
**36**	*ent*-beyer-15-en-17-ol	*E.* *australe*, *E.* *pictum*, *E.* *argentinum*, *E.* *rotundifolium*, *E.* *zambesiacum*, *E.* *monogynum*	[5, 20, 31, 38]
**37**	*ent*-beyer-15-en-17,19-diol	*E.* *monogynum*	[38]
**38**	*ent*-beyer-15-en-1*α*-ol	*E.* *australe*	[5]
**39**	*ent*-beyer-15-en-12*β*-ol	*E.* *zambesiacum*	[31]
**40**	*ent*-beyer-15-en-7-one	*E.* *argentinum*	[31]
**41**	*ent*-2-hydroxybeyer-2,15-dien-1-one	*E.* *australe*	[5, 24]
**42**	*ent*-2,17-dihydroxybeyer-2,15-dien-1-one	*E.* *australe*, *E.* *pictum*, *E.* *microphyllum*, *E.* *argentinum*	[5, 20, 31]
**43**	*ent*-2,19-dihydroxybeyer-2,15-dien-1-one	*E.* *australe*, *E.* *microphyllum*	
**44**	*ent*-15,16-epoxy-beyer-1-one	*E.* *australe*	[5, 24]
**45**	*ent*-15,16-epoxy-beyerene	*E.* *zambesiacum*, *E.* *monogynum*	[31, 38, 39]
**46**	erythroxylol A epoxide	*E.* *monogynum*	[38]
**47**	erythroxylol A acetate epoxide	*E.* *monogynum*	[38]
**48**	4*β*-hydroxy-18-norhibaene	*E.* *monogynum*	[38]
**49**	4*α*-hydroxy-18-norhibaene	*E.* *monogynum*	[38]
**50**	*ent-*beyer-15-en-19-al	*E.* *monogynum*	[40]
**51**	*ent*-17-hydroxybeyer-15-en-1-one	*E.* *australe*, *E.* *pictum*, *E.* *areolatum*, *E.* *argentinum*, *E.* *microphyllum*, *E.* *rotundifolium*	[5, 20, 31]
**52**	*ent*-2*α*,17-dihydroxybeyer-15-en-1-one	*E.* *pictum*, *E.* *microphyllum*, *E.* *rotundifolium*	[20, 31]
**53**	*ent*-2*α*,19-dihydroxybeyer-15-en-1-one	*E.* *australe*, *E.* *betulaceum*, *E.* *microphyllum*	[5, 31, 32]
**54**	*ent*-beyer-15-en-1-one	*E.* *australe*, *E.* *argentinum*, *E.* *rotundifolium*, *E.* *zambesiacum*	[5, 24, 31]
**55**	*ent*-2*α*-hydroxybeyer-15-en-1-one	*E.* *australe*	[24]
**56**	*ent*-19-hydroxybeyer-15-en-1-one	*E.* *australe*	[5]
**57**	*ent*-1*α*-hydroxybeyer-15-en-2-one	*E.* *australe*	[5]
**58**	*ent*-1*α*,17-dihydroxybeyer-15-en-2-one	*E.* *pictum*, *E.* *rotundifolium*	[20, 31]
**59**	*ent*-1*α*,19-dihydroxybeyer-15-en-2-one	*E.* *microphyllum*	[31]
**60**	isoatisirene	*E.* *monogynum*	[27]
**61**	atisirene	*E.* *monogynum*	[27]
**62**	*ent*-kauran-16-ol	*E.* *pictum*, *E.* *australe*	[5, 20]
**63**	*ent*-kauran-16,17-diol	*E.* *rotundifolium*, *E.* *pictum*	[20, 31]
**64**	erythroxylisin A	*E.* *barbatum*	[41]
**65**	erythroxylisin B	*E.* *barbatum*	[41]
**66**	*ent*-12*β*-hydroxy-kaur-16-en-19-al	*E.* *suberosum*	[33]
**67**	methylent-7*β*,15*α*-dihydroxy-kaur-16-en-19-oate	*E.* *suberosum*	[33]
**68**	(+)-devadarene	*E.* *monogynum*	[27]
**69**	*ent-*devadarane-15*ξ*,16-diol	*E.* *monogynum*, *E.* *barbatum*, *E.* *macrocarpum*, *E.* *pictum*, *E.* *sideroxyloides*	[20, 31] [35, 36]
**70**	*ent*-devadaran-l*α*,11*α*,15*ξ*,16-tetrol	*E.* *australe*, *E.* *areolatum*, *E.* *sideroxyloides*	[5, 20, 31]
**71**	*ent*-l*α*-acetoxydevadaran-11*α*,15*ξ*,16-triol	*E.* *areolatum*	[31]
**72**	triol Q	*E.* *monogynum*	[43]
**73**	*ent*-11*α*-acetoxy-devadarane-15*ξ*,16-diol	*E.* *barbatum*	[35]
**74**	*ent*-devadarane-11*α*,15*ξ*,16-triol	*E.* *barbatum*	[35]
**75**	*ent*-devadarane-7*β*,15*ξ*,16-triol	*E.* *barbatum*, *E.* *monogynum*	[35, 36]
**76**	ryanodanol	*E.* *passerinum*, *E.* *nummularia*	[13]
**77**	14-*O*-methyl-ryanodanol	*E.* *passerinum*	[13]

### Triterpenoids

**Fig. 5:**
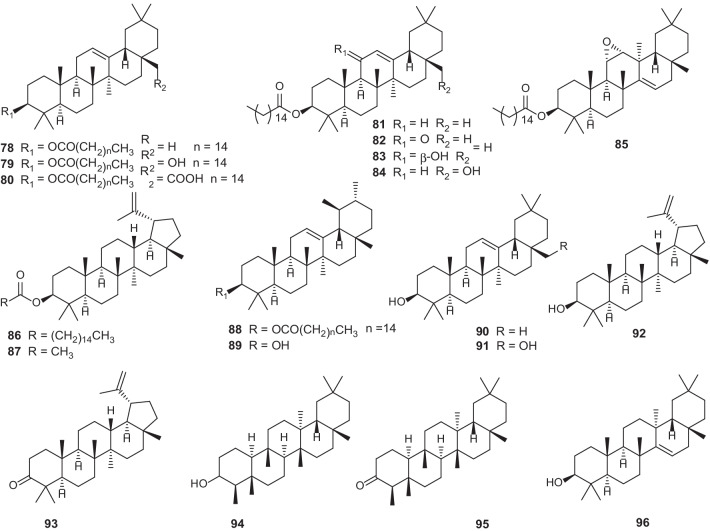
Chemical structures of triterpenoids (**78–96**) found in *Erythroxylum* plants

To date, a total of 19 triterpenoids have been identified in *Erythroxylum* plants (Fig. 5; Table 5), ten of which are fatty acid esters of triterpenes (**78–86, 88**) from *E.* *nummularia* [44], *E.* *leal*-*costae* [45], *E.* *rimosum* [46] or *E.* *passerinum* [47]. Lupenyl acetate (**87**) [45], *α*-amyrin (**89**) [46], *β*-amyrin (**90**) [44, 46, 47] and erythrodiol (**91**) [47] are other four triterpenoids found in *E.* *leal*-*costae*, *E.* *nummularia*, *E.* *rimosum* or *E.* *passerinum* (Fig. 5; Table 5). Besides, recent studies reported five triterpenes (**92–96**) from *E.* *ovalifolium* [14], *E.* *daphnites* [48] or *E.* *macrocalyx* [49]. Interestingly, all the triterpenoids identified in this genus are pentacyclic triterpenes.

**Table 5:** Triterpenoids isolated from *Erythroxylum* plants

No.	Compounds name	Plant source	Refs.
**78**	*β*-amyrin palmitate and stearate	*E.* *nummularia*, *E.* *rimosum*	[44, 46]
**79**	erythrodiol palmitate and stearate	*E.* *nummularia*	[44]
**80**	oleanolic acid	*E.* *nummularia*	[44]
**81**	*β*-amyrin palmitate	*E.* *passerinum*, *E.* *leal*-*costae*	[45, 47]
**82**	3*β*-hydroxy-11-oxo-olean-12-enylpalmitate	*E.* *passerinum*	[47]
**83**	3*β*,11*β*-dihydroxy-olean-12-enyl palmitate	*E.* *passerinum*	[47]
**84**	3*β*,28-dihydroxy-olean-12-enyl palmitate	*E.* *passerinum*	[47]
**85**	3*β-*hydroxy-11,12-epoxy–friedoolean-14-enyl palmitate	*E.* *passerinum*	[47]
**86**	lupenyl palmitate	*E.* *leal*-*costae*	[45]
**87**	lupenyl acetate	*E.* *leal*-*costae*	[45]
**88**	*α*-amyrin esters	*E.* *rimosum*	[46]
**89**	*α*-amyrin	*E.* *rimosum*	[46]
**90**	*β*-amyrin	*E.* *nummularia*, *E.* *passerinum*, *E.* *rimosum*	[44, 46, 47]
**91**	erythrodiol	*E.* *passerinum*	[47]
**92**	lupeol	*E.* *macrocalyx*, *E.* *ovalifolium*	[14, 49]
**93**	lupenone	*E.* *macrocalyx,* *E.* *daphnites*	[48, 49]
**94**	friedelanol	*E.* *daphnites*	[48]
**95**	friedelan-3-one	*E.* *daphnites*, *E.* *subsessile*	[14, 48]
**96**	taraxerol	*E.* *macrocalyx*	[49]

### Alkaloids

**Fig. 6:**
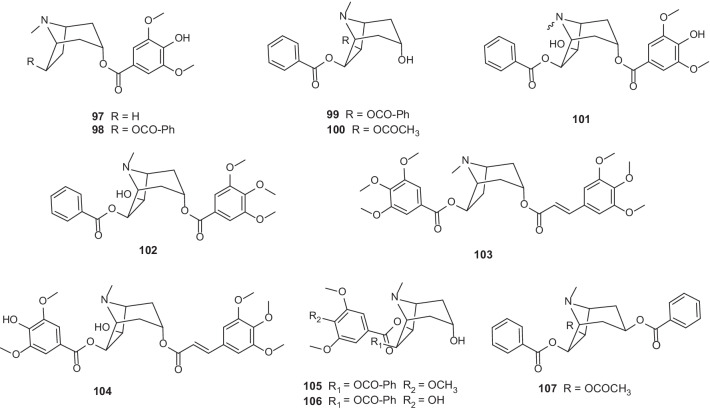
Chemical structures of TAs (**97–107**) found in *Erythroxylum* plants after 2010

TAs are alkaloids with a tropane skeleton (8-azabicyclo[3.2.1]octane). As characteristic alkaloids widely distributed in *Erythroxylum* species, TAs exhibit a range of pharmacological activities like vasorelaxation [50], antiproliferative [49], anesthesia [51], antimicrobial and anticancer [12]. In 2010, a fascinating review by Oliveira et al. [6] comprehensively summarized structures of 186 TAs found in 35 species of *Erythroxylum*. As an update, we here found 11 more new TAs reported in studies since then (Fig. 6; Table 6). Among these newly identified TAs, two members were isolated from *E.* *pungens* (**97**) [52] and *E.* *caatingae* (**98**) [53], respectively; 6*β*,7*β*-dibenzoyloxytropan-3*α*-ol (**99**) was obtained from *E.* *subsessile* [54]; 7*β*-acetoxy-6*β*-benzoyloxy-3*α*-hydroxytropane (**100**) was isolated from the twigs of *E.* *macrocalyx* [49]; six members named as erythrobezerrines A-F (**101–106**) were isolated from the stem bark of *E.* *bezerrae* [55]; and 7*β*-acetoxy-3*β*,6*β*-dibenzoyloxytropane (**107**) was isolated from the leaves of *E.* *rimosum* [46]. Previous studies have also reported the isolation of non-TA alkaloids by GC–MS analysis [56–58]. However, since most of them were potential precursors or side products of TA biosynthetic pathway [56], we will not include them here. Readers interested in the details of these compounds are referred to the review by Brachet Anne and coworkers [56].

**Table 6:** Alkaloids isolated from *Erythroxylum* plants

No.	Compounds name	Plant source	Refs.
**97**	pungencine	*E.* *pungens*	[52]
**98**	6*β*-benzoyloxy-3*α*[(4-hydroxy-3,5-dimethoxybenzoyl) oxy] tropane	*E.* *caatingae*	[53]
**99**	6*β*,7*β*-dibenzoyloxytropan-3*α*-ol	*E.* *subsessile*	[54]
**100**	7*β*-acetoxy-6*β*-benzoyloxy-3*α*-hydroxytropane	*E.* *macrocalyx*	[49]
**101**	erythrobezerrine A	*E.* *bezerrae*	[55]
**102**	erythrobezerrine B	*E.* *bezerrae*	[55]
**103**	erythrobezerrine C	*E.* *bezerrae*	[55]
**104**	erythrobezerrine D	*E.* *bezerrae*	[55]
**105**	erythrobezerrine E	*E.* *bezerrae*	[55]
**106**	erythrobezerrine F	*E.* *bezerrae*	[55]
**107**	7*β*-acetoxy-3*β*,6*β*-dibenzoyloxytropane	*E.* *rimosum*	[46]

### Flavonoids

**Fig. 7:**
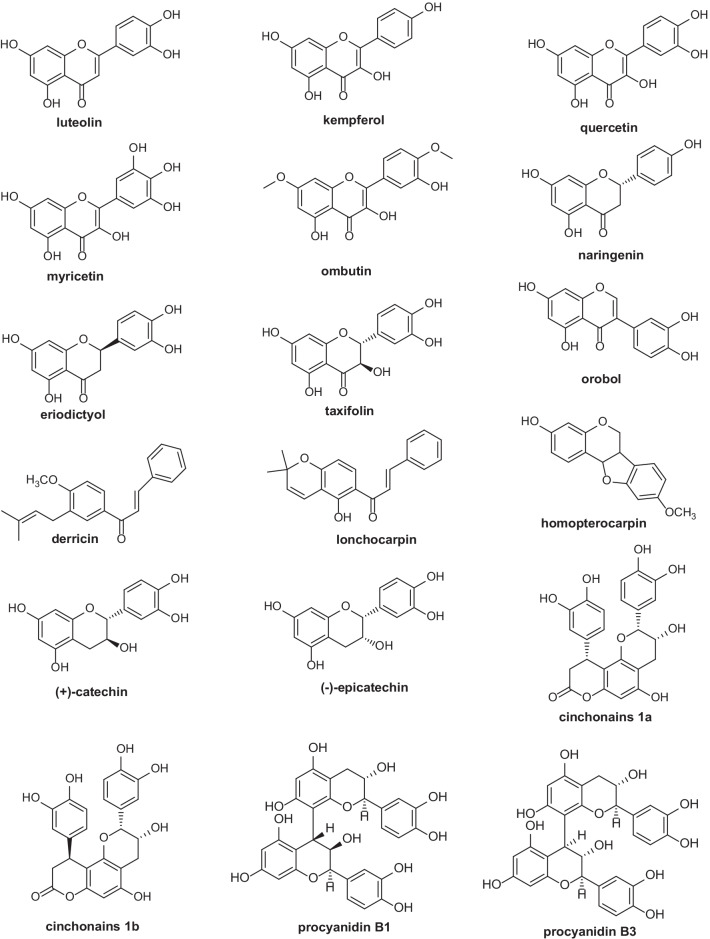
Chemical structures of flavonoid aglycones found in *Erythroxylum* plants

Flavonoids are a large and complex group of constituents found in almost all plants. Flavonoid variation in thirteen species of *Erythroxylum* has been studied systematically by Plowman et al. in 1988 [59]. They found kaempferol, ombuin (7,4ʹ-dimethylquercetin), and quercetin were predominant flavonoid aglycones in *Erythroxylum* plants analyzed. Besides, Johnson and coworkers [60–65], based on their work on flavonoids profiles of six species or variants and flavonoids that had been reported in *Erythroxylum*, proposed that some unique flavonoids could be used as chemotaxonomic markers for taxon. Overall, flavonoid aglycones in *Erythroxylum* mainly consist of quercetin, ombuin, fisetin, kaempferol, epicatechin, eriodictyol and taxifolin. In addition to these, isoflavone, isoflavanone and other flavone derivatives were also found in *Erythroxylum*. Chemical structures of flavonoid aglycones that have been found in *Erythroxylum* plants were summarized and presented in Fig. 7. Moreover, the major glycosides of these flavonoids include mono-glucosyl-rhamnosyls and dirhamnosyl-glucosides, as well as mono-galactosyl and mono-arabinosyl. In total, 73 flavonoids from 37 species of *Erythroxylum* have been studied (Table 7), though some structures lack NMR data support in the literature.

**Table 7:** Flavonoids isolated from *Erythroxylum* plants

No.	Compounds name	Plant source	Refs.
**108**	ombuin	*E.* *nummularia*	[44]
**109**	ombuin-3-*O*-rutinoside	*E.* *alaternifolium*, *E.* *campestre*, *E.* *barbatum*, *E.* *argentinum*, *E.* *tenue*, *E.* *daphnites*, *E.* *loefgrenii*, *E.* *engleri*, *E.* *cuneifolium*, *E.* *lucidum*, *E.* *pruinosum*, *E.* *pulchrum*, *E.* *squamatum*, *E.* *subracemosum*, *E.* *subrotundum*, *E.* *vacciniifolium*, *E.* *novogranatense*	[59, 66–71]
**110**	ombuin-3-*O*-rutinoside-5-*O*-glucoside	*E.* *argentinum*, *E.* *cuneifolium*, *E.* *pulchrum*, *E.* *macrocalyx*	[49, 67, 70, 71]
**111**	quercetin	*E.* *rimosum*, *E.* *lucidum*, *E.* *suberosum*, *E.* *ovalifolium*	[14, 46, 59]
**112**	quercetin-3-*O*-rutinoside	*E.* *argentinum*, *E.* *cambodianum*, *E.* *alaternifolium*, *E.* *engleri*, *E.* *loefgrenii*, *E.* *leal*-*costae*, *E.* *lucidum*, *E.* *pruinosum*, *E.* *coca*, *E.* *suberosum*, *E.* *subracemosum*, *E.* *ovalifolium*, *E.* *rufum*, *E.* *ulei*	[4, 10, 14, 45, 59, 66–68, 72, 73]
**113**	quercetin-3-*O*-rhamnoside	*E.* *argentinum*, *E.* *cuneatum*, *E.* *ulei*, *E.* *rufum*, *E.* *subsessile*, *E.* *daphnites*, *E.* *loefgrenii*, *E.* *lucidum*, *E.* *leal*-*costae*, *E.* *pruinosum*, *E.* *pulchrum*, *E.* *suberosum*, *E.* *vacciniifolium*, *E.* *laurifolium*, *E.* *macrocarpum*, *E.* *hypericifolium*	[14, 45, 59, 67, 68, 73–75]
**114**	quercetin-3-*O*-glucoside	*E.* *daphnites*, *E.* *loefgrenii*, *E.* *pruinosum*, *E.* *rimosum*, *E.* *rufum*, *E.* *squamatum*, *E.* *suberosum*, *E.* *subracemosum*, *E.* *ulei*, *E.* *coca*, *E.* *vacciniifolium*, *E.* *nummularia*, *E.* *laurifolium*, *E.* *macrocarpum*, *E.* *hypericifolium*	[4, 10, 44, 46, 59, 67, 73, 75]
**115**	quercetin-3-*O*-arabinoside	*E.* *campestre*, *E.* *cuspidifolium*, *E.* *pruinosum*, *E.* *rufum*, *E.* *rimosum*, *E.* *ulei*, *E.* *suberosum*, *E.* *vacciniifolium*	[46, 59, 73]
**116**	quercetin-3-*O*-xyloside	*E.* *campestre*, *E.* *rufum*, *E.* *ulei*, *E.* *vacciniifolium*	[59, 73]
**117**	quercetin-3-*O*-galactoside	*E.* *rufum*, *E.* *rimosum*, *E.* *ulei*, *E.* *suberosum*	[46, 59, 73]
**118**	quercetin-3-*O*-glucosylxyloside	*E.* *campestre*, *E.* *squamatum*, *E.* *suberosum*	[59]
**119**	quercetin-3-*O*-glucosylarabinoside	*E.* *campestre*, *E.* *suberosum*	[59]
**120**	quercetin-3-*O*-glucosylglucoside	*E.* *vacciniifolium*, *E.* *ulei*	[59, 73]
**121**	quercetin-3-*O*-rhamnoside-7-*O*-glucoside	*E.* *vacciniifolium*, *E.* *australe*	[59, 65]
**122**	quercetin-3,7-*O*-dirhamnoside	*E.* *vacciniifolium*	[59]
**123**	quercetin-4′,3-di-*O*-rhamnoside	*E.* *coca* var. *ipadu*	[64]
**124**	quercetin-4′,7-di-*O*-rhamnoside	*E.* *ulei*	[63]
**125**	kaempferol	*E.* *rimosum*	[46]
**126**	kaempferol-3-*O*-glucoside	*E.* *barbatum*, *E.* *loefgrenii*, *E.* *rufum*, *E.* *squamatum*, *E.* *ulei*, *E.* *suberosum*, *E.* *subracemosum*, *E.* *tenue*, *E.* *vacciniifolium*	[59, 73]
**127**	kaempferol-3-*O*-arabinoside	*E.* *cuspidifolium*, *E.* *daphnites* *E.* *suberosum*, *E.* *vacciniifolium*, *E.* *rufum*, *E.* *ulei*, *E.* *rimosum*	[46, 59, 73]
**128**	kaempferol-3-*O*-rhamnoside	*E.* *loefgrenii*, *E.* *pruinosum*, *E.* *rufum*, *E.* *subsessile*, *E.* *tenue*, *E.* *ulei*, *E.* *vacciniifolium*	[14, 54, 59, 73]
**129**	kaempferol-3-*O*-galactoside	*E.* *rufum*, *E.* *ulei*, *E.* *vacciniifolium*	[59, 73]
**130**	kaempferol-3-*O*-xyloside	*E.* *rufum*, *E.* *suberosum*, *E.* *ulei*, *E.* *vacciniifolium*	[59, 73]
**131**	kaempferol-3-*O*-glucosylxyloside	*E.* *barbatum*, *E.* *campestre*	[59]
**132**	kaempferol-3-*O*-rutinoside	*E.* *rufum*, *E.* *ulei*, *E.* *subracemosum*, *E.* *tenue*	[59, 73]
**133**	kaempferol-3-*O*-arabinofuranoside	*E.* *rimosum*	[46]
**134**	kaempferol-3-*O*-glucoside-7-*O*-rhamnoside	*E.* *cuneifolium*, *E.* *tenue*, *E.* *vacciniifolium*	[59, 71]
**135**	kaempferol-3-*O*-arabinoside-7-*O*-rhamnoside	*E.* *vacciniifolium*	[59]
**136**	kaempferol-3-*O*-rhamnoside-7-*O*-galactoside	*E.* *novogranatense.* var. *novogranatense*	[60]
**137**	kaempferol-3,7-*O*-dirhamnoside	*E.* *cuneifolium*	[71]
**138**	kaempferol-3-*O*-triacetylrhamnoside-7-*O*-triacetylgalactoside	*E.* *novogranatense.* var. *novogranatense*	[60]
**139**	kaempferol-4′-ethoxy-7-*O*-galactoside	*E.* *novogranatense.* var. *novogranatense*	[60]
**140**	kaempferol-4′-*O*-rhamnosylglucoside	*E.* *coca* var*.* *ipadu*	[64]
**141**	kaempferol-3,4′-di-*O*-rhamnoside	*E.* *coca* var*.* *ipadu*	[64]
**142**	kaempferol-3-*O*-rutin-7-*O*-rhamnoside	*E.* *coca* var*.* *ipadu*	[64]
**143**	taxifolin	*E.* *ulei*	[73]
**144**	taxifolin-3,4′-di-*O*-rhamnoside	*E.* *coca* var*.* *ipadu*	[64]
**145**	taxifolin-3,7,4′-tri-*O*-rhamnoside	*E.* *coca* var*.* *ipadu*	[64]
**146**	eriodictyol-7-*O*-rhamnoside	*E.* *coca* var*.* *ipadu*, *E.* *australe*	[64, 65]
**147**	eriodictyol-3′-ethoxy-4′-*O*-rhamnoside	*E.* *coca* var*.* *ipadu*	[64]
**148**	eriodictyol-3′-ethoxy-4′-*O*-acetylrhamnoside	*E.* *coca* var*.* *coca*	[60]
**149**	eriodictyol-7-*O*-acetylrhamnoside	*E.* *coca* var*.* *coca*	[60]
**150**	eriodictyol-7-*O*-triacetylrhamnoside	*E.* *coca* var*.* *coca*	[60]
**151**	eriodictyol-3′-ethoxy-7-*O*-acetylrhamnoside	*E.* *coca* var*.* *coca*	[60]
**152**	eriodictyol-3′-ethoxy-7-*O*-triacetylrhamnoside	*E.* *coca* var*.* *coca*	[60]
**153**	eriodictyol-3′,4′-di-ethoxy-7-*O*-acetylrhamnoside	*E.* *coca* var*.* *coca*	[60]
**154**	luteolin-3′-ethoxy-4′-H-3-*O*-rhamnoside	*E.* *novogranatense.* var*.* *novogranatense*	[60]
**155**	luteolin-3′-OH-4′-H-3-*O*-triacetylrhamnoside	*E.* *novogranatense.* var*.* *novogranatense*	[60]
**156**	luteolin-8-*O*-rhamnoside	*E.* *leal*-*costae*	[45]
**157**	luteolin-6-*O*-rhamnoside	*E.* *leal*-*costae*	[45]
**158**	myricetin-3-*O*-glucoside	*E.* *ulei*	[73]
**159**	naringenin-7-*O*-glucoside	*E.* *ulei*	[73]
**160**	dihydro-orobol-4′-*O*-dirhamnoside	*E.* *australe*	[65]
**161**	dihydro-orobol-7-methoxy-5-*O*-rhamnoside	*E.* *australe*	[65]
**162**	dihydro-orobol-7-*O*-glucoside-3′-*O*-rhamnoside	*E.* *australe*	[65]
**163**	dihydro-orobol-5-dehydroxy-7,3′-di-*O*-glucoside	*E.* *australe*	[65]
**164**	dihydro-orobol-2-methyl-3′-*O*-rhamnoside	*E.* *australe*	[65]
**165**	orobol-2,5′-dihydroxy-7-*O*-dirhamnoside	*E.* *ulei*	[63]
**166**	orobol-3′-dehydroxy-4-*O*-glucoside-7-*O*-dirhamnoside	*E.* *ulei*	[63]
**167**	orobol-2-hydroxy-7-*O*-dirhamnoside	*E.* *ulei*	[63]
**168**	dihydro-orobol-2-methyl-4′-*O*-galactoside-7-*O*-dirhamoside	*E.* *ulei*	[63]
**169**	dihydro-orobol-2-methyl-4′-*O*-galactoside-7-*O*-rhamoside	*E.* *ulei*	[63]
**170**	derricin	*E.* *barbatum*	[76]
**171**	medicarpin	*E.* *barbatum*	[76]
**172**	lonchocarpin	*E.* *barbatum*	[76]
**173**	homopterocarpin	*E.* *barbatum*	[76]
**174**	( +)-catechin	*E.* *cambodianum*, *E.* *cuneatum*, *E.* *rimosum*, *E.* *suberosum*	[10, 46, 72, 74]
**175**	(+)-catechin-3-*O*-*α*-rhamnopyranoside	*E.* *novogranatense*	[69]
**176**	(−)-epicatechin	*E.* *cambodianum*, *E.* *rimosum*, *E.* *suberosum*, *E.* *leal*-*costae*	[10, 45, 46, 72]
**177**	procyanidin B1	*E.* *novogranatense*	[69]
**178**	procyanidin B3	*E.* *novogranatense*	[69]
**179**	cinchonains la	*E.* *catuaba*	[11]
**180**	cinchonains lb	*E.* *catuaba*	[11]

### Other constituents

**Fig. 8:**
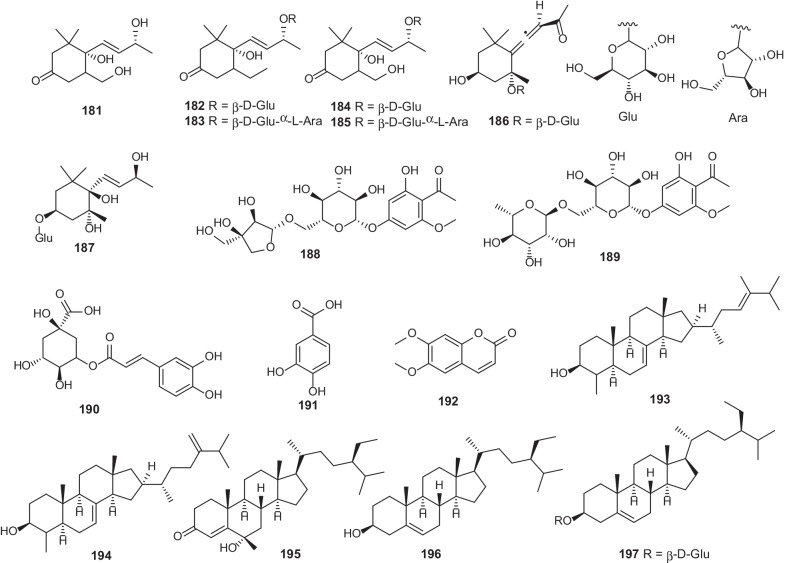
Chemical structures of other constituents (**181–197**) found in *Erythroxylum* plants

Norisoprenoid compounds (megastigmanes, **181–187**) have been characterized in *E.* *cuneatum* [74] and *E.* *cambodianum* [72] by Kanchanapoom et al. (Fig. 8; Table 8). Phenolic derivatives and their glycosides were also obtained (Fig. 8; Table 8), which include two acetophenone diglycosides (**188–189**) isolated from *E.* *cambodianum* [72], neochlorogenic acid (**190**) and protocatechuic acid (**191**) extracted from *E.* *lucidum* [68], and scoparon (**192**) yielded from *E.* *barbatum* [76]. Additionally, five steroids (**193–197**) have been identified in this genus according to the previous studies [35, 44, 46–48, 76, 77] (Fig. 8; Table 8). Importantly, compounds **193** and **194** showed significant anti-oxidant and anti-glycation activities in vitro [77].

**Table 8:** Other constituents isolated from *Erythroxylum* plants

No.	Compounds name	Plant source	Refs.
**181**	apocynol B	*E.* *cuneatum*	[74]
**182**	(6*S*,9*R*)-roseoside	*E.* *cuneatum*	[74]
**183**	vomifoliol-9-*O*-arabinofuranosyl-glucopyranoside	*E.* *cuneatum*	[74]
**184**	inamoside	*E.* *cuneatum*	[74]
**185**	cuneatoside	*E.* *cuneatum*	[74]
**186**	citroside A	*E.* *cuneatum*, *E.* *cambodianum*	[72, 74]
**187**	(3*S*,5*R*,6*R*,7*E*,9*S*)-megastigman-7-ene-3,5,6,9-tetrol-3-*O*-*β*-glucopyranoside	*E.* *cambodianum*	[72]
**188**	erythroxylosides A	*E.* *cambodianum*	[72]
**189**	erythroxylosides B	*E.* *cambodianum*	[72]
**190**	neochlorogenic acid	*E.* *lucidum*	[68]
**191**	protocatechuic acid	*E.* *lucidum*	[68]
**192**	scoparon	*E.* *barbatum*	[76]
**193**	4-methyl ergosta-7,23-dien-3*β*-ol	*E.* *monogynum*	[77]
**194**	4-methyl ergosta-7,24(28)-dien-3*β*-ol	*E.* *monogynum*	[77]
**195**	steroids procesterol	*E.* *barbatum*	[76]
**196**	*β*-sitosterol	*E.* *barbatum*, *E.* *daphnites*, *E.* *rimosum*, *E.* *nummularia*, *E.* *passerinum*	[44, 46–48, 76]
**197**	*β*-sitosterol-*O*-glucoside	*E.* *barbatum*	[35]

## Biological activities of natural products in *Erythroxylum*

### Bioactivities of diterpenes, triterpenes and sterols

Pharmacological investigation of diterpenes isolated from *Erythroxylum* species are still scarce despite the large resource found. Diterpene 14-*O*-methyl-ryanodanol (**77**) showed insecticidal activity against *Aedes* *aegypti* larvae [13], as well as a dose-dependent cytotoxic effect to astrocytes (GL-15 cell line) [78]. Cytotoxicity activities against five tumor cell lines of devadarane derivatives (**69**, **73–75**) were also investigated, but no activity was observed [35]. Exploring and evaluating bioactivities of the numerous diterpenoids found in *Erythroxylum* species will be essential for further effective utilization of these natural product resources in this genus. For triterpenes, compounds **93–95** were major constituents of the hexane extract of *E.* *daphnites* leaves which showed a cytotoxic effect against SCC-9 oral squamous cell carcinoma cell line [48]. Additionally, sterols (**193**, **194**) isolated from *E.* *monogynum* possess good anti-oxidant and anti-glycation activities [77]. Additionally, although a large number of flavonoids have been found in *Erythroxylum* species, these compounds are not specifically distributed in this genus. Readers interested in the details of bioactive flavonoids are referred to the review by Shashank Kumar and coworkers [79].

### Bioactivities of TAs

In Erythroxylaceae family, TAs specially occur in species of *Erythroxylum*. Until now, a total of 197 TAs compounds have been characterized in *Erythroxylum* plants. There are plenty of researches on pharmacology activities of TAs in *Erythroxylum*, especially cocaine. Ophthalmologist Carl Koller first demonstrated the ability of cocaine to induce local anesthesia in eyes [8]. Later, it was extended to dentistry, urology, laryngology and other fields as a local anaesthetic [80]. In addition, a review by Drake [51] highlighted that cocaine could act as a psychomotor stimulant and also showed toxicity in coabuse and overdoses. Cocaine acts on the mesolimbic dopamine system whose origins begins in the ventral tegmental area and projects to the nucleus accumbens, the amygdala, the hippocampus, and the prefrontal cortex, resulting in a higher concentration of dopamine release into the nucleus accumbens and prefrontal cortex [81]. Previous study also showed that acute cocaine at a dose used by cocaine abusers for recreational purposes induced large increases in intracellular calcium in the cortex of the rat brain and the mechanism were related to the local anesthetic actions of cocaine and not its sympathomimetic effects [82]. The cardiovascular mitochondrial dysfunction induced by cocaine is involved in the mechanisms of oxidative stress [83]. Also, Ca^2+^/calmodulin-dependent protein kinase II and inhibitory G-protein coupled receptor signaling are involved in the mechanism of the effect of cocaine- and amphetamine-regulated transcript in cocaine reward [84]. There are a number of excellent reviews on the bioactivity, toxicity, and biological mechanisms of cocaine [84–88], and therefore we will not repeat more details here. Additionally, for cocaine-producing *Erythroxylum* plants, cocaine could function as a natural insecticide to protect the leaves [89].

Pervilleine A (reviewed in ref. [6]) from *E.* *pervillei* demonstrated weak nonspecific anticholinergic and vascular antiadrenergic activities [90]. Catuabine B and 3*α*,6*β*-dibenzoyloxytropane from *E.* *vaccinifolium* [53] (reviewed in ref. [6]) showed antimicrobial activity on gram-positive bacteria and fungi [12]. It has also been demonstrated that the *E.* *cuneatum* leaf alkaloid extract possessed both antioxidative and anti-inflammatory properties [9]. Among the reported biological activities of TAs, cytotoxicity is also noticeable. Araújo Neto et al. [91] summarized the cytotoxic activity of 21 species of *Erythroxylum* against 45 different cell lines and found the species with presence of disubstituted TAs had the highest cytotoxic potentials. Recently, a newly identified tropane alkaloid (6*β*-benzoyloxy-3*α*[(4-hydroxy-3,5-dimethoxybenzoyloxy] tropane) (**98**) was demonstrated to possess high antiproliferative activity on liver hepatocellular carcinoma cells (HepG2) with IC_50_ value of 3.66 μg mL^−1^. Meanwhile, it showed no cytotoxicity on human lymphoblast cell line [49]. In addition, erythrobezerrine C (**103**) showed moderate cytotoxicity activity on HCT-116 and NCI-H460, with IC_50_ values of 3.38 and 5.43 μM, respectively [55]. TAs with antimicrobial [12] and diuretic [92, 93] activities have also been reported. In 1984, Novak [94] reported the bioactivities of TAs from *E.* *coca* and *E.* *novogranatense* contained stimulant activity, inhibiting effect on dopamine uptake, and cholinolytic action.

### Bioactivities of crude extracts

In addition to research on single compound, many studies have been carried out on the biological activities of crude extracts of *Erythroxylum* plants (Table 9). *E.* *monogynum* is rich in alkaloids and diterpenes. In 2019, Dhanunjaya et al. [95] summarized that crude extracts of this species had multiple bioactivities, such as antioxidant, antihyperlipidemic, antidiabetic, antiplasmodial and hepatoprotective. Particularly, leaf and bark extracts of *E.* *delagoense*, *E.* *emarginatum*, or *E.* *pictum*, showed great antibacterial activities [96]. Ethanolic extract obtained from the roots of *E.* *pungens* could induce dose-dependent hypotension and tachycardia in conscious rats, as well as vasorelaxation in mesenteric artery ring preparations in vitro [50]*.* Ethanolic extract of *E.* *caatingae* has a relaxant effect on ovine cervical contractions [97]. Besides, low-polarity fractions of this species showed significantly high cytotoxicity activity against the NCI-H292, HEp-2 and K562 cell lines [12]. Furthermore, acetone/water (70/30, v/v) extract of *E.* *macrocarpum* is a significant inhibitor of acetylcholinesterase [98]. Hydroalcoholic extracts of *E.* *areolatum* or *E.* *confusum* showed antiherpetic activity [99]. For the antitumor activity, when mice were treated with different doses of methanol extract of *E.* *caatingae*, a significant reduction in their tumor weight was observed [53]. Moreover, extracts of *E.* *minutifolium* or *E.* *confusum* showed hepatoprotective effects [100]. Crude extracts, fractions, or isolated products of *E.* *ovalifolium* or *E.* *subsessile* were demonstrated to inhibit toxic effects of the snake (*Lachesis* *muta*) venom, providing a new strategy for antivenom treatment [14].

**Table 9:** Biological activities of crude extracts of *Erythroxylum* plants

Plant source	Extract Source	Crude extracts	Pharmocological activities	Refs.
*E.* *monogynum*	Leaves	Chloroform	Antidiabetic	[95]
		Ethanolic	Hepatoprotective effects; Nephroprotective effects	[95, 101]
		Aqueous	Antimicrobial; Antioxidant	[95]
		Methanol	Antiplasmodial; Cytotoxicity	[95, 102]
*E.* *delagoense*	Leaves and bark	Acetonic; Methanol; Aqueous	Antibacterial	[96]
*E.* *emarginatum*	leaves and stems	Acetonic; Methanol; Aqueous	Antibacterial	[96]
*E.* *pictum*	Leaves and stems	Acetonic; Methanol; Aqueous	Antibacterial	[96]
*E.* *pungens*	Roots	Ethanolic	Vasorelaxant	[50]
*E.* *caatingae*	Leaves	Ethanolic	Myorelaxing effect on smooth muscle tissue	[97, 103]
	Stems	Methanol;	Antimicrobial activity;	[12, 53]
		Low-polarity fractions	Cytotoxicity	
*E.* *macrocarpum*	Leaves	Acetone/water (70/30, v/v)	Acetylcholinesterase inhibition	[98]
*E.* *areolatum*	Leaves	Hydroalcoholic	Antiherpetic activity	[99]
*E.* *confusum*	Leaves	Hydroalcoholic	Antiherpetic activity	[99]
*E.* *minutifolium*	Leaves	n-Hexane	Hepatoprotective effects	[100]
*E.* *confusum*	Leaves	n-Hexane	Hepatoprotective effects	[100]
*E.* *ovalifolium*	Stems	Ethanolic	Neutralize toxicity of snake venom	[14]
*E.* *subsessile*	Stems	Ethanolic	Neutralize toxicity of snake venom	[14]
*E.* *daphnites*	Leaves	n-Hexane	Anti-proliferative effects	[48]

## Conclusions and prospecting

Based on the current progress in phytochemistry of the *Erythroxylum* [6], there is no doubt that TAs are the largest class of compounds found in this genus (197 of 383 compounds). In the past years, their remarkable pharmacological activities have made this class of compounds receive more attention than others [49, 52, 104]. However, many other types of active compounds have been found in *Erythroxylum* along with the broadening and deepening of phytochemical research. A summary of the structure and distribution of these compounds is essential for in-depth understanding and utilization of plant resources of this genus. Based on the literature, a total of 383 compounds from *Erythroxylum* have been reported, among which only 186 tropane alkaloids have been reviewed in 2010. In this review, we summarized all remained 197 compounds characterized from 53 *Erythroxylum* species from 1960 to 2021, including 11 skeleton-types of diterpenes (**1**–**77**) isolated from 18 *Erythroxylum* species, 19 triterpenoids obtained from 8 *Erythroxylum* species, 11 TAs found in 6 species after 2010, 73 flavonoids from 37 *Erythroxylum* species, and 17 other constituents (norisoprenoids, phenolic derivatives and their glycosides, and steroids). Among these compounds, most diterpenes were isolated from the timber or roots of the plants, triterpenes were identified from aerial organs, flavonoids were distributed in leaves or branches, while others had no obvious tissue- or organ-specific distributions. Significant biological activities, including anaesthetic [8], antioxidative [9, 10], anti-inflammatory [9], cytotoxic [11], anticancer [12], and insecticidal activities [13], as well as neutralization of snake venom [14], have been demonstrated for isolated products or crude extracts from some species of *Erythroxylum*. However, potential activity of most compounds is still unknown. In-depth biological activity studies on compounds obtained will be the basis for exploring potential medicinal resources in this genus. Additionally, some of the diterpenes were suggested to serve as the defensive components to protect the *Erythroxylum* plants from herbivores, pathogens, or other environmental challenges. Therefore, they could be used as potential bioinsecticides in agriculture in the future.

**Fig. 9:**
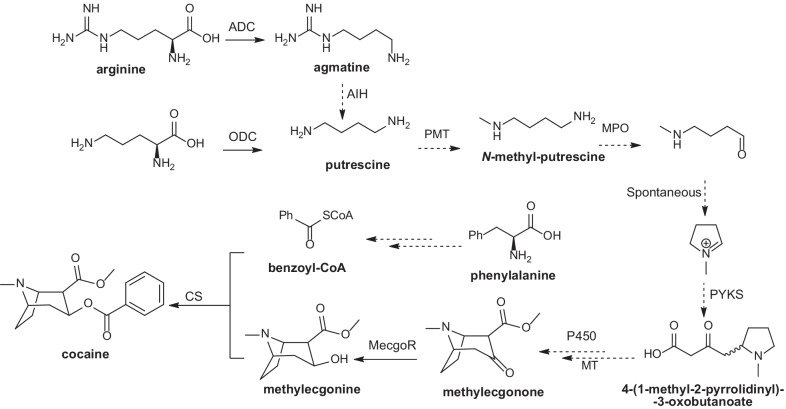
The proposed cocaine biosynthesis pathway in *E.* *coca*. The following enzymes are depicted in the figure above: ADC (arginine decarboxylase), ODC (ornithine decarboxylase), AIH (armatine iminohydrolase), PMT (putrescine methyltransferase), MPO (*N*-methylputrescine oxidase), PYKS (pyrrolidine ketide synthase), P450 (cytochrome 450), MT (methyltransferase), MecgoR (methylecgonone reductase), CS (cocaine synthase)

Elucidation of natural product biosynthetic pathways has been proved to be highly useful for natural products discovery, structure identification and subsequent heterologous synthesis. In *Erythroxylum* plants, TAs and diterpenes are representative phytoconstituents. Biochemists and molecular biologists have long sought to identify the biosynthetic pathways of TAs, especially cocaine, through isotope labeled precursor feeding studies and gene cloning and characterization [105–110]. As a result, incomplete biosynthetic route of cocaine starting from arginine and ornithine and passing through putrescine, methylecgonone, and methylecgonine has been established [7, 110] (Fig. 9), though further studies are still essential to elucidate the missing steps. Studies focusing on the biosynthesis pathway of diterpenes in *Erythroxylum* plants have not been reported till now. However, the kaurene-type (Fig. 1i) diterpene synthase that is responsible for the formation of *ent*-kaurene, the universal biosynthetic intermediate of gibberellin, has been identified in many other plants [111–113]. Besides, *ent*-beyerene synthase, which is the key diterpene cyclase required for generating *ent*-beyerene type diterpenes (Fig. 1h), has been characterized in monocotyledonous rice (*Oryza* *sativa* L.) [114]. Still, much more researches needed to be done for better understanding the biosynthetic mechanisms and diversity of diterpenes identified in *Erythroxylum*.
